# Mechanical Twinning is a Correlated Dynamic Process

**DOI:** 10.1038/s41598-019-42317-4

**Published:** 2019-04-08

**Authors:** A. Vinogradov, E. Agletdinov, D. Merson

**Affiliations:** 1 Department of Mechanical and Industrial Engineering, Norwegian University of Technology – NTNU, Trondheim, 7491 Norway; 2 Institute of Advanced Technologies, Togliatti State University, Togliatti, 445020 Russia

## Abstract

Plastic deformation and fracture of materials is accompanied by generation of elastic wave transients known as acoustic emissions (AE). A novel complex methodology combining the statistical analysis of distributions of time intervals between the successive AE events, and the unsupervised cluster analysis of the time series is proposed to test for possible correlations between emitting sources and to highlight their intrinsic dynamics. Aiming at revealing the essential temporal features of the twinning and dislocation dynamics, the proposed methodology was applied to the AE signals produced during plastic deformation in a magnesium alloy where both primary deformation modes - dislocation slip and twinning - operate concurrently. It has been undoubtedly demonstrated that the mechanical twinning belongs to a class of non-Poisson processes having a memory of the past in the millisecond range. As opposes to the correlated behaviour of twins, it was shown that the dislocation slip falls into the category of Poisson processes caused by independent sources.

## Introduction

## Motivation and Methodology

Plastic deformation in many metals and alloys with the hexagonal close-packed (HCP) lattice is mediated by a combination of dislocation slip and mechanical twinning - two primary mechanisms of plastic flow at low homologous temperatures. Local stress fields associated with twinning are pivotal in the deformation behaviour and fracture but are extremely difficult to characterize experimentally^1^, particularly in dynamics whereby the twins interact with each other and/or with dislocations. Their mutual activity and interactions control the work hardening behaviour of magnesium-based alloys. Both these mechanisms generate elastic waves commonly referred to as acoustic emissions (AE)^2^ reflecting the specific dynamics of the sources and allowing for unique access to real-time information on underlying deformation processes. Bearing a strong similarity to seismology^3–5^, the AE technique, combined with multi-parametric descriptive statistical analysis and pattern recognition methods^6^, provides a powerful means of correlating the activity of elementary deformation processes - slip and twinning with the strain hardening behaviour^7^. The influence of various metallurgical factors (texture, grain size, and solute content) on AE and underlying twinning and dislocation slip behaviour under different loading modes (tension or compression) has been investigated in significant detail^8–12^. Similarly to pure FCC and BCC metals under uniaxial tensile loading, the AE activity in HCP Mg and its alloys commences at the very beginning of loading, peaks shortly after yielding and decays gradually with strain^13,14^). Due to its inherent sensitivity to dynamic processes only, the AE technique is an ideal tool for probing intermittency of the plastic flow, which is spatially heterogeneous and temporally fluctuating in nature due to a discrete character of dislocations and their avalanches^15,16^. AE stemming from dislocation mechanisms such as breaking away from unevenly distributed pinning points, motion between randomly spaced obstacles, escaping to a free surface at random locations along the gauge length, etc. involves a relatively large number of cooperatively moving dislocation segments and appears as an autoregressive stochastic process represented by a train of low-amplitude transients^17,18^. The question remains if the same applies to mechanical twins arising as a result of the strongly correlated motion of twinning dislocations, and generating powerful AE bursts with the amplitude proportional to the length and velocity of the nucleating twin^19,20^. The random AE time series can be fully characterised statistically in the multivariate space of features the shortest list of which includes the amplitude of transients and their inter-arrival times. In this way, the AE time series can be regarded as a point process. In fact, point processes can represent a wide variety of behaviours. The two most distinct types of underlying dynamics comprise a history-independent behaviour of autonomous and uncorrelated sources, and a history-dependent dynamics of interconnected, statistically correlated events. Thus, the temporal complexity of the collective dynamics of defects in solids during plastic flow is reflected in AE and can be made explicit through an adequate data analysis. While the statistical properties and the non-linear dynamics of dislocation avalanches arising during both smooth and jerky plastic flow have been intensively studied using the AE amplitude distributions^16,21–24^, the statistical behaviour of mechanical twins has not been explored to that extent^25^.

The Poisson random process model is the first choice to assess the dynamics of the emitting sources and possible correlations between them. It has indisputably set the basis for modelling efforts in many fields. It has become a widespread tool in materials science and geoscience, aiming at characterising strongly localised deformation and fracture evolving on different scales in time and in different patterns ranging from the nanometre-wide shear band in metallic glasses^26,27^ to rock mechanics^4,28,29^ and mega-earthquakes^30,31^. The Poisson distribution dominates the stochastic modelling philosophy whenever the sources are considered independent and it constitutes the grounds for a family of derivative renewal models accounting for deviations from the ideal Poissonian distribution due to the memory of the past and the correlation between the sources^32–34^ or due to inhomogeneity of the time-dependent intensity λ which itself might be a stochastic process (c.f. Cox or Pólya processes and their generalisations)^35,36^.

**Figure 1:**
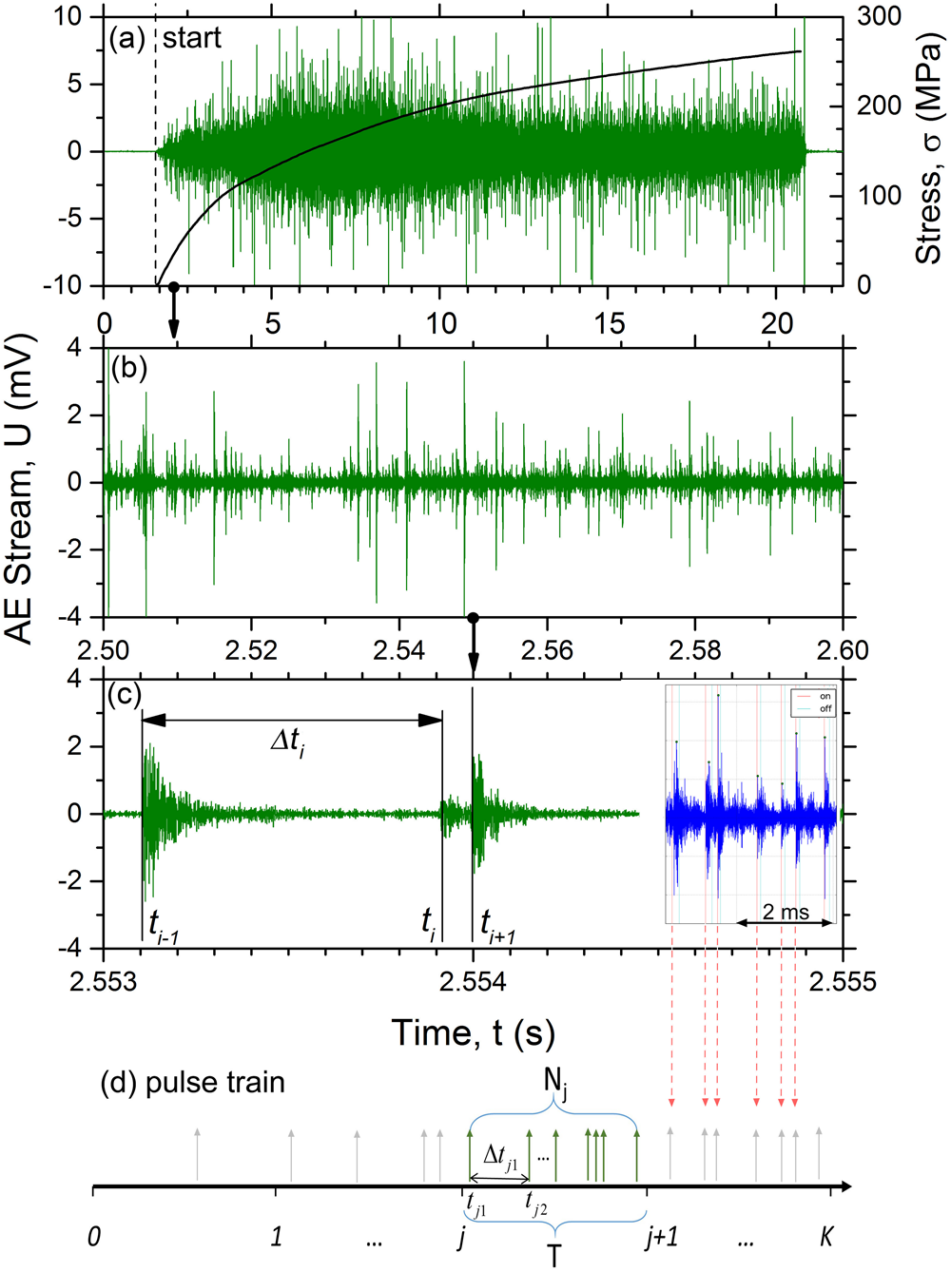
Acoustic emission data synchronised with the tension loading curve of monotonic tension test of the ZK60 Mg-alloy: (**a**) a whole dataset (**b**,**c**) magnified fragments from (**a**) showing the AE time series at different temporal resolutions. Dashed line in (**a**) indicates the beginning of loading; vertical arrows show approximately the intervals where fragments (**b**,**c**) were selected. The inset in (**c**) shows the fragment of AE stream and the results of application of the ϕ signal detector; (**d**) is a schematics of AE representation as a point process characterised solely by the inter-arrival times between the successive events detected by the *ϕ*-algorithm.

For the assessment of possible correlations in the behaviour of emitting defects, the AE time series is characterised by a single parameter – a set of arrival times of events $\{{t}_{0},{t}_{1}\,\mathrm{...}\,{t}_{i}\,\mathrm{...}\,{t}_{N}\}$ as is commonly adopted for point processes. In practice, it is more convenient to investigate the distribution of time intervals between successive events ${\rm{\Delta }}{t}_{i}={t}_{i+1}-{t}_{i}$, Fig. 1. Assuming that the sources of local stress relaxation resulting in the AE events are independent, one should observe a Poisson time series defined as a train of Dirac’s δ-impulses $\delta (t-{t}_{k})$ with amplitudes *U*_*k*_

1 $$A(t)=\sum _{k=1}^{N}{U}_{k}\delta (t-{t}_{k})$$

and the inter-arrival time intervals Δ*t*_*i*_ obeying an exponential distribution with the probability density function

2 $$\rho ({\rm{\Delta }}t)=\frac{1}{\overline{{\rm{\Delta }}t}}\exp (-\,{\rm{\Delta }}t/\overline{{\rm{\Delta }}t})=\lambda \exp (\,-\,\lambda {\rm{\Delta }}t)$$

where $\overline{{\rm{\Delta }}t}$ is the average (over the time interval *T*) time interval between the pulses in a time-series (or its chosen fragment) and $\lambda =1/\overline{{\rm{\Delta }}t}$ is the average intensity (count rate or activity) of the pulse flow on the chosen time interval *T*.

Using the proposed robust statistical approach based on a blend of independent statistical procedures applied to the AE time series recorded during twinning-mediated plastic deformation of the magnesium alloy ZK60, we endeavour to reveal (and make explicit) correlations in the collective dynamics of mechanical twins, and to demonstrate that twinning is a history-dependent stochastic process.

## Results and Data Analysis

Twin nucleation in HCP crystals occurs primarily from grain boundaries^1,37^ by simultaneous glide of multiple twinning dislocations^38^. This cooperative dislocation motion produces strong AE transients rising to peak values within few microseconds since the twins nucleate very fast, at a sizable fraction of the velocity of sound^39^, due to the fact that the stress required for twin propagation is lower than that for twin nucleation. Therefore, once nucleated, the twin propagates through the grain in both longitudinal and lateral directions until another twin variant, the opposite grain boundary or immobile dislocations impede twin movement. It has been well understood that AE results from rapid twin nucleation while the slow lateral twin growth (thickening) does not produce any measurable AE^20,40–42^. The mutual interactions between twins can, therefore, be elucidated by the use of statistical analysis of transients in AE time series.

**Figure 2:**
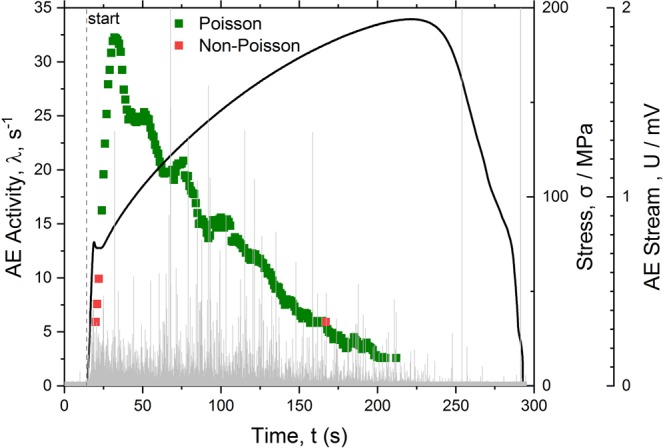
The loading curve (solid line) and the concurrent behaviour of the AE activity *λ* (squares) in pure α-Fe during monotonic tensile deformation. The light grey plot on the background corresponds to the raw streaming data. The green colour marks the fragments of the AE stream where the distribution of the inter-arrival times agrees with the exponential Poisson distribution $\rho ({\rm{\Delta }}t)$, Eq. (2), while the red colour corresponds to the realisations with the non-Poisson behaviour according to the ${\chi }^{2}$ goodness-of-fit test with the confidence level 0.05.

**Figure 3:**
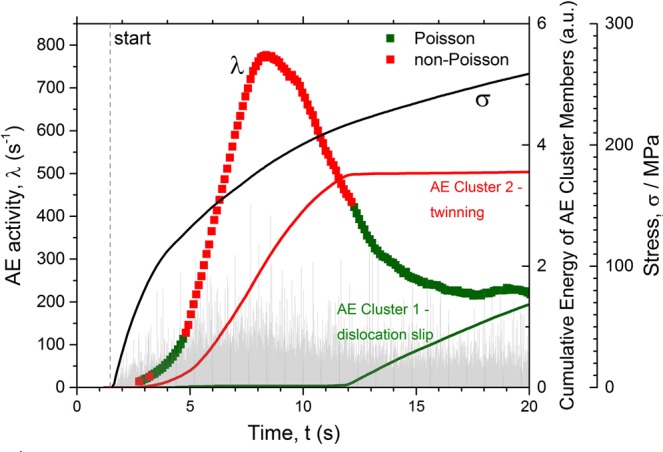
The loading curve (solid line) and the concurrent behaviour of the AE activity *λ* (squares) in the ZK60 Mg alloy during monotonic tensile deformation. The light grey plot on the background corresponds to initial raw data shown in Fig. 1. The solid lines represent the results of the independent AE cluster analysis revealing two deformation mechanisms corresponding to dislocation slip and twinning. An excellent agreement between the results of two independent techniques has to be stressed.

Raw data representing the typical AE time series recorded in the course of tensile testing of the Mg-alloy ZK60 are shown in Fig. 1 at different scales revealing the discrete nature of AE signals mixed with continuous background noise. Similarly to most structural metals (c.f. Fig. 2 for well-annealed α-Fe), AE exhibits a broad peak after yielding, Fig. 1a, which then reduces as strain hardening proceeds. Figure 1b and c show that the AE stream in Mg alloy consists of a large number of randomly appearing well-separated transient signals with broadly varying amplitudes. The AE arrival times $\{{t}_{0},{t}_{1},\,\mathrm{...},\,{t}_{N}\}$ obtained from the continuously recorded AE signal constitute the point process as illustrated schematically in Fig. 1c,d. Since the properties of any random process are fully determined by a probability density function (PDF) of a descriptive random variable, the PDF $\rho ({\rm{\Delta }}t)$ is obtained for each *j-th* realisation, and the statistical goodness-of-fit χ^2^ test is applied to probe the agreement between the inter-arrival times Δ*t* and the Poisson distribution (2) with the count rate ${\lambda }_{j}={N}_{j}/T$ for each realisation $j\in [1,K]$. Main findings of the statistical analysis are shown in Figs 2 and 3 for pure α-Fe and ZK60 alloy, respectively (for comparison, α-Fe is taken here as a representative of a class of materials deforming solely by intermittent dislocation slip mechanisms while the Mg-based alloy demonstrates profuse twinning). In these figures, the AE intensity $\lambda$ is superimposed with the loading diagram and the original raw AE stream shown on a background.

**Figure 4:**
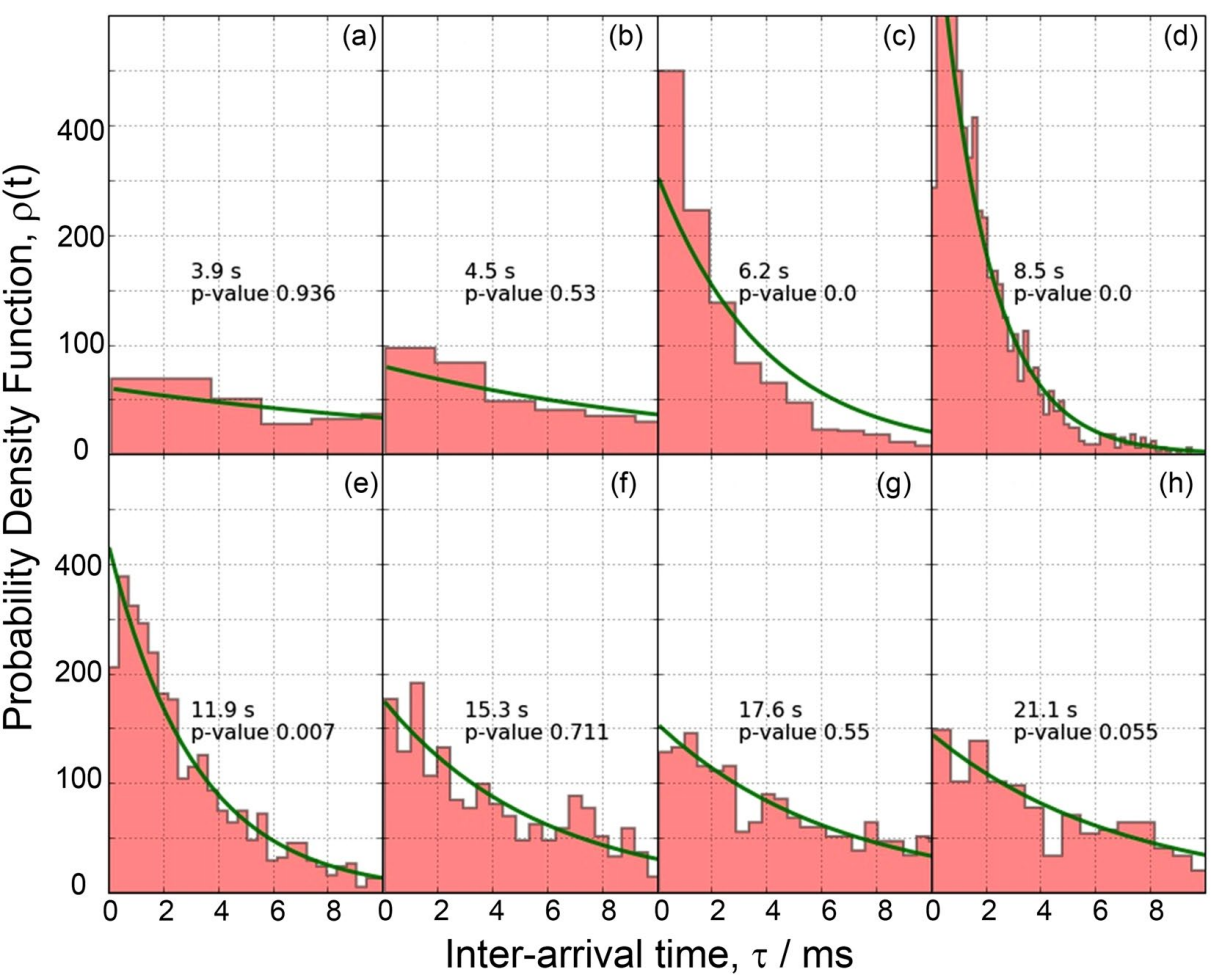
The evolution of the distribution function of time intervals between successive events in the AE time series corresponding to Fig. 3 at different time intervals $j\in [1,K]$ shown in each sub-figure. The histograms show the experimental probability density function of the inter-arrival times. The solid lines represent the exponential Poisson distributions expected for given *λ* according to Eq. (2) (notice that it is not a fitting curve).

It is instructive to notice that two generic types of AE behaviour – Poisson and non-Poisson, arise depending on the underlying deformation mechanism as highlighted in these figures. The individual slip lines, such as those reflecting the dislocation-mediated AE response in pure well annealed Fe with mean grain size of 150 μm, Fig. 2, emerge on a free surface as a result of the collective dislocation behaviour which can be resolved by the modern AE technique revealing separated transients masked in the continuous noise-like signal^17^. Even though every elementary slip event involves the correlated motion of a large number of atoms on a microscale, during strain hardening, numerous slip lines appear sporadically and independently in different grains of polycrystalline aggregates^18^. The Poisson type AE is therefore reasonably expected unless the collective effects in dislocation ensembles come into force in the form of coarse slip bands or plastic instabilities^43,44^. This can be nicely seen in Fig. 2, where the Poisson type AE is confirmed over the entire uniform hardening range in excellent agreement with the assumption made in^18^, while the non-Poisson (correlated) behaviour is seen during the Lüders band propagation stage where the correlated dislocation behaviour is naturally anticipated. The collective dynamics of twins is notably different. Typical randomly chosen experimental distributions of inter-arrival times observed on different stages of deformation of the alloy ZK60 are shown in Fig. 4 where the expected ideal theoretical Poisson distributions with the count rate *λ* (same as that observed experimentally for a given sample) according to Eq. (1) are also displayed by solid lines. The χ^2^ test shows that at the very beginning of deformation, AE commences as the Poisson process, i.e. good agreement exists between the observed and expected exponential distributions of waiting times. The same can be said with high confidence for the mature stage of deformation (ca.12–20 s) before necking and fracture sets in. Similarly to Fig. 2, this is illustrated schematically in Fig. 3 by green squares. As opposes to this, the χ^2^ Pearson’s *p*-value being much less than the 0.05 significance level suggests that we cannot accept the null hypothesis and that there is a significant difference between the observed and the expected Poisson distribution from approximately 5 to 12 s of the deformation process. This is highlighted by red on the intensity curve in Fig. 3. We should notice that this time interval corresponds to the appearance of the broad peak of AE intensity which is most commonly associated with the dominance of twinning in plastic deformation of Mg and its alloys^41,45,46^.

To confirm the results of the ${\chi }^{2}$ hypothesis testing and to get a deeper insight into the nature of the event–event correlations, the independent *Bi*-test^47^ was used. Originally proposed in astrophysics as a method for probing local correlations in time series, this test has been successfully used to characterise the statistics of the local fracture events during compression of shale rocks^48^. As opposes to the Pearson’s the ${\chi }^{2}$ test, it is valid also for strongly non-stationary processes. The application of the *Bi*-test with a floating window has fully confirmed the results of the ${\chi }^{2}$ test. In particular, in the realisations where the hypothesis of the exponential distribution of time intervals is rejected, the *Bi*-test also suggests the presence of local correlations (the hypothesis of uniformity of *B* is rejected). Therefore, the consistency of the results of both independent tests can only lead one to conclude undeniably that there are local correlations in the studied stream of AE events.

As a matter of fact, two remarkably different statistics characterise AE data on different loading stages, and this has to be rationalised on the basis of operating deformation mechanisms. The observed significant deviation in the distribution of AE emitting sources from the Poisson-like behaviour is a prominent result since it expressly provides the evidence for the existence of a sort of statistical correlation between the defects underlying plastic flow in Mg and its alloys. To draw any conclusions on the nature of this correlation, one has (i) to identify the deformation mechanisms responsible for the observed correlations (e.g. dislocation slip, twinning, the interplay between these two, etc.), and (ii) to clarify its character by specifying an appropriate model of the non-Poisson process describing experimental data. To address the first problem, we employ the signal categorisation technique capable of distinguishing between different AE sources (see Methods for details).

**Figure 5:**
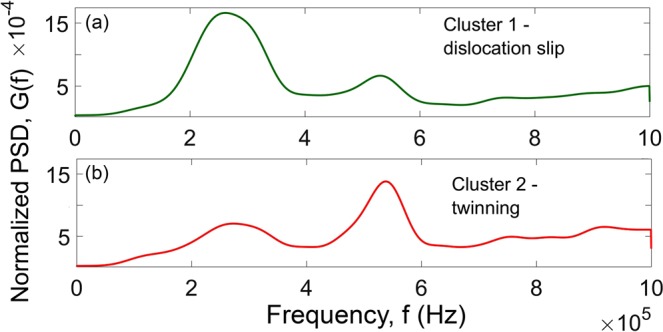
The centroids of normalised AE PSD functions characterising two main AE clusters which kinetics is represented in Fig. 3: (**a**) the average PSD for Cluster 1 corresponding to dislocation slip and (**b**) the average PSD for Cluster 2 corresponding to mechanical twinning.

Since the plastic strain associated with a single mechanical twin shooting through the grain is small^49^, a part of plastic strain has to be accommodated by dislocation slip, and both mechanisms co-exist in the course of plastic deformation. This implies that virtually any realisation of the AE random process contains contributions from both mechanisms and it is important to distinguish the respective contributions. This has been accomplished with aid from a quantitative statistical cluster analysis of AE time series in Mg and its alloys^7,19,50,51^. This analysis is based on the premise that dislocation slip and twinning are significantly different in their dynamics and therefore they generate AE with different waveforms. Specifically, continuous low-amplitude waveforms produced by dislocation slip can be compared to high amplitude transients for twinning^7,40^. Using the *adaptive sequential k-means* (ASK) AE signal clusterisation algorithm^6^ (see also^7,50,52,53^ for details), the realisations of 4k samples with similar Fourier power spectral density (PSD) were grouped in the same category, while the realisations with statistically different PSDs were disjoined. This non-supervised procedure reveals that the AE signals fall naturally into two primary categories with statistically different shapes of power spectra, as demonstrated in Fig. 5, representing the cluster centroids (mean PSD) for each AE source. Evidently, both clusters differ in the relative fraction of low and high-frequency components in their PSD. The noise-like Cluster 1 is characterised by the prevailing lower frequency content and low amplitude AE, which is typically associated with the dominance of dislocation slip in materials. Cluster 2 is composed of high-frequency transient signals with high peak amplitudes assuming from the preponderance of mechanical twinning. Thus, following the similar reasoning provided in^7,50,53^, Cluster 1 was associated with dislocation slip while Cluster 2 was attributed to twinning which appeared to be predominant from 5 to 12 s of deformation, Fig. 3. One should bear in mind that, in fact, as deformation proceeds, both deformation mechanisms - dislocation slip and twinning - co-exist contributing to the resultant AE throughout the entire loading process. The ASK algorithm makes it possible to identify the predominant mechanisms underlying the particular stages of strain hardening, and the contributions of each mechanism to the released AE energy have been found to be different depending on the strain, c.f. also^7,50^.

The remarkable result is that both independent data processing methods (the analysis of the distributions of waiting times between transients and the cluster analysis) show a strikingly good agreement on the time domains where the AE time series has distinct statistical features, Fig. 3: the non-AE Poisson behaviour is observed when the twinning appears as a dominant deformation mode, whereas when dislocation slip prevails as the predominant mechanism of plastic deformation (after of 12 s deformation), the AE obeys the Poisson statistics. In other words, while the dislocation motion during uniform plastic flow can be regarded as a random process of independent, uncorrelated sources, the mechanical twinning is a stochastic process with intrinsic correlations or some memory of the past.

The most intriguing finding of the present work is the deviation of the experimental AE time-series generated by mechanical twinning from the ideal Poisson process. The observed memory of the past can be short or long resulting in a conceptually different understanding of the background process. The short memory processes are grouped around a Poissonian background process with possible foreground dependants triggered by Poisson-distributed precursors while emphasising the long memory in the temporal and spatial evolution leads to self-organised criticality, power-law behaviour and long tail distributions. In order to get a deeper insight into the nature of this phenomenon, one has to identify a model of the point process which adequately describes experimental data.

A broad variety of candidates belonging to different classes of stochastic processes can be considered as potentially appropriate for characterising the observed correlations. Generally speaking, a temporal point process is specified through its *conditional intensity function* ${\lambda }^{\ast }{(t)}_{{H}_{t}}$ defined as^35,54,55^;

3 $${\lambda }^{\ast }{(t)}_{{H}_{t}}=\frac{{f}^{\ast }(t)}{1-{F}^{\ast }(t)}$$

where the subscript *H*_*t*_ denotes symbolically the history of the process to time *t*, containing the list of arrival times (i.e. ${\lambda }^{\ast }$ at the moment generally depends on all *t*_*i*_ < *t*), ${f}^{\ast }(t)$ is the conditional probability density function and ${F}^{\ast }(t)$ is the cumulative distribution function (CDF). Here the term “conditional” indicates that the process depends on the past and evolves. Hence, the conditional intensity function appears as a convenient means characterising how the present depends on the past in an evolutionary point process. For the Poisson process, the conditional intensity function is equal to the history-independent intensity $\lambda (t)$ entering Eq. (2), i.e. ${\lambda }^{\ast }{(t)}_{{H}_{t}}=\lambda (t)$.

Let us recall that a class of point processes, which are referred to as *renewal processes*, is defined via independent identically distributed intervals. The Poisson processes (both homogeneous with $\lambda (t)=const$ and heterogeneous with $\lambda (t)\ne const$) belong to this class. Considering its alternative with the short memory of the past, the so-called Hawkes process introduced in 1971^34,56^ is admittedly one of the best-known candidates. In this process, the parent events follow a Poisson process, and multiple generations of descendants are triggered, relative to their parent. In this sense, the Hawkes process can be regarded as the “autoregressive” point process^35^. Modifications of this model have become the predominant models for earthquake aftershocks (ETAS) as well as for many other applications. Unlike Markovian processes disregarding the history of the process, the idea of the Hawkes process is centred on the premise that the memory of the past is an important intrinsic property of the generating process. Thus, the Hawkes process is the simplest extension of the Poisson point process, in which past events influence future events in such a way that the occurrence of any event increased the probability of further events occurring. It is therefore defined as a point process whose conditional intensity is a function of the past data ${H}_{t}$^34,56^:

4 $${\lambda }^{\ast }{(t)}_{{H}_{t}}=\mu +\sum _{{t}_{i} < t}a{e}^{-C(t-{t}_{i})}$$

where $\mu \in (0,\infty )$ is the positively defined deterministic count rate providing a Poisson base level for the process, and $\varphi (t-{t}_{i})=a{e}^{-C(t-{t}_{i})}$ is the triggering/self-excitation kernel, which regulates the intensity at time *t* that is made by an event that occurs in the past at a previous time ${t}_{i}$ (both *a* and *C* are the positive constants)^57^. According to Eq. (4), each time a new event arrives, the conditional intensity increases sharply by a factor of *a* and then decreases exponentially back towards the *μ* value of the background Poisson process. Here *a* determines the magnitude of growth while *С* controls the rate of decay. One can notice that the conditional intensity (4) at the time moment *t* depends, indeed, on the whole time history *t*_*i*_ < *t*, and the set of model parameters includes *μ*, *a*, and *C*: ${\rm{\Theta }}=\{\mu ,a,C\}$.

**Figure 6:**
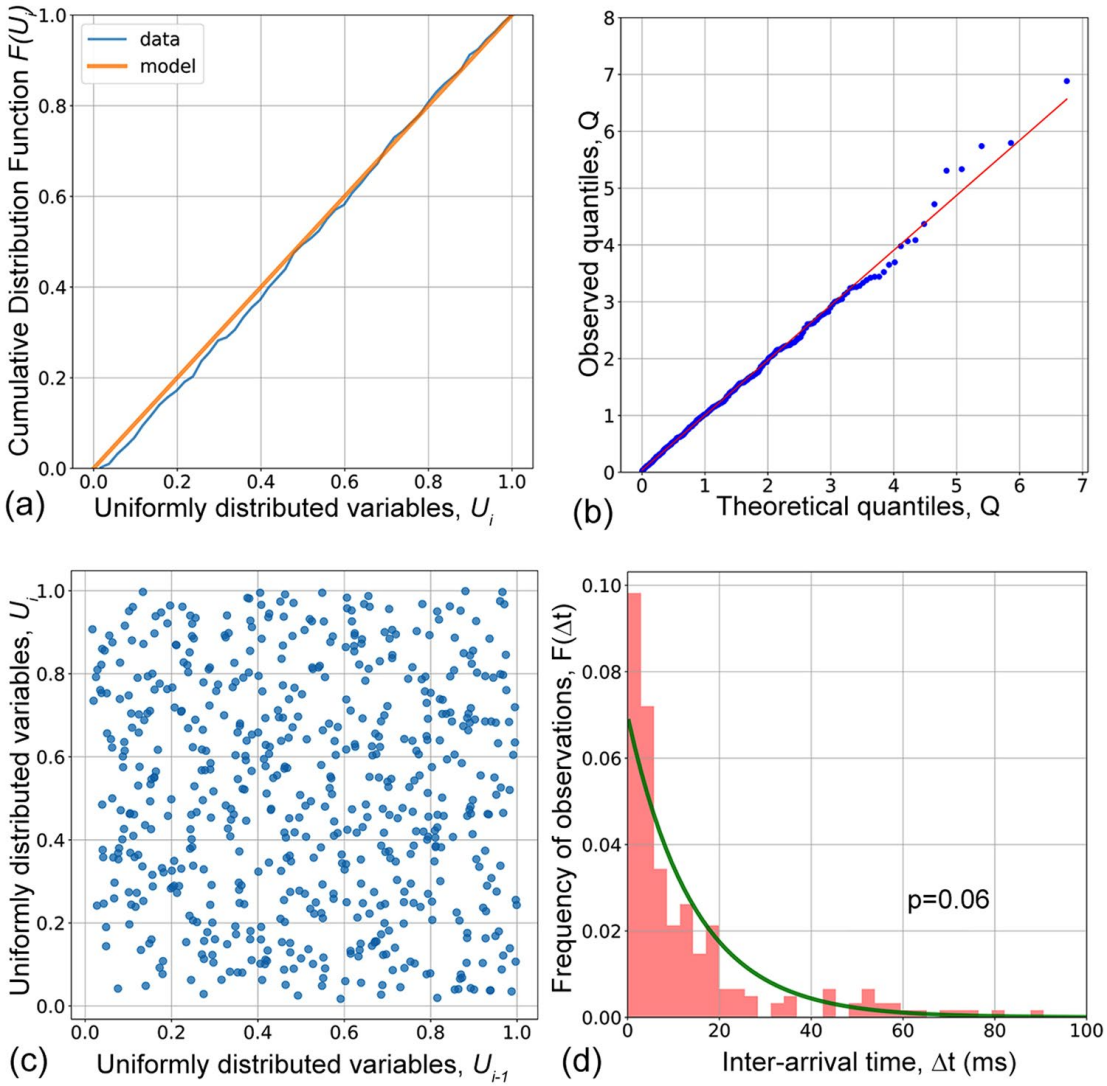
Results of probing the Hawkes model on the actual AE dataset corresponding to the regime of twinning dominance from 5 to 12 s in Fig. 1 for the ZK60 Mg alloy: statistical plots representing the CDF for the *U*-variables (**a**), Q-Q plot (**b**), the scatter plot of ${U}_{i}$ vs. ${U}_{i-1}$ variables (**c**) and the inter-arrival time-frequency histogram with the superimposed Poisson model exponent (**d**).
(ii) the Q-Q plot, Fig. 6b, showing the quantile of the distribution of ${\tau }^{\ast }$ in the rescaled AE time series against the quantile of the $\exp (\,-\,{\tau }^{\ast })$ distribution strongly suggests that both distributions are essentially the same;
(iii) the points on the correlation plot ${U}_{i}-{U}_{i-1}$, Fig. 6c, are almost evenly distributed;
(iv) the results of testing the null hypothesis that the distribution of inter-arrival times of the transformed process corresponds to the exponential distribution $\exp (\,-\,{\tau }^{\ast })$ with the *p-*value greater than the criterial 0.05 while the initial distribution of inter-arrival times, Fig. 6d, cannot be considered Poissonian according to the same testing procedure.

The Hawkes model was tested for consistency with experimental AE data using the statistical procedure described in Methods section. The dataset shown in Fig. 1 was taken as a typical example for illustration. Assuming the Hawkes model, the optimal parameters determined by the Nelder-Mead solver are given as ${{\rm{\Theta }}}_{opt}=\{\mu ,\,a,\,C\}=\{170,\,14.04,\,9.86\}$. The further detailed statistical analysis performed according to the scheme outlined above confirms that the Hawkes model provides an adequate first-order fit to the investigated time series:

(i) the cumulative distribution function, Fig. 6a, of the doubly transformed AE point process is linear for its *U* values;

In conclusion, the behaviour of AE produced primarily by mechanical twinning exhibits a short time correlation, and this memory of the past can be adequately described by the Hawkes type self-excited point process.

## Discussion

Virtually any modelling, including the probabilistic ones, inevitably exaggerates some aspects of the phenomenon of interest while disregarding others. An adequate model is supposed to highlight the essential aspects and shadow the less important details. In this sense, the Hawkes point process model of the observed AE behaviour is the first order approximation that adequately captures the most salient feature of mechanical twinning evolution in Mg and its alloys – it is a correlated process having the memory of the past not only at the initiation phase generating the AE pulse, but also during collective dynamics of twin ensembles generating pulse trains. It also suggests that this memory is caused by the influence of twinning events on the immediately following events on the sub-millisecond time scale. Figure 4(c–e) illustrate clearly that the largest deviation of the inter-arrival times from the exponential Poisson distribution is seen in the short time domain below 1 ms.

**Figure 7:**
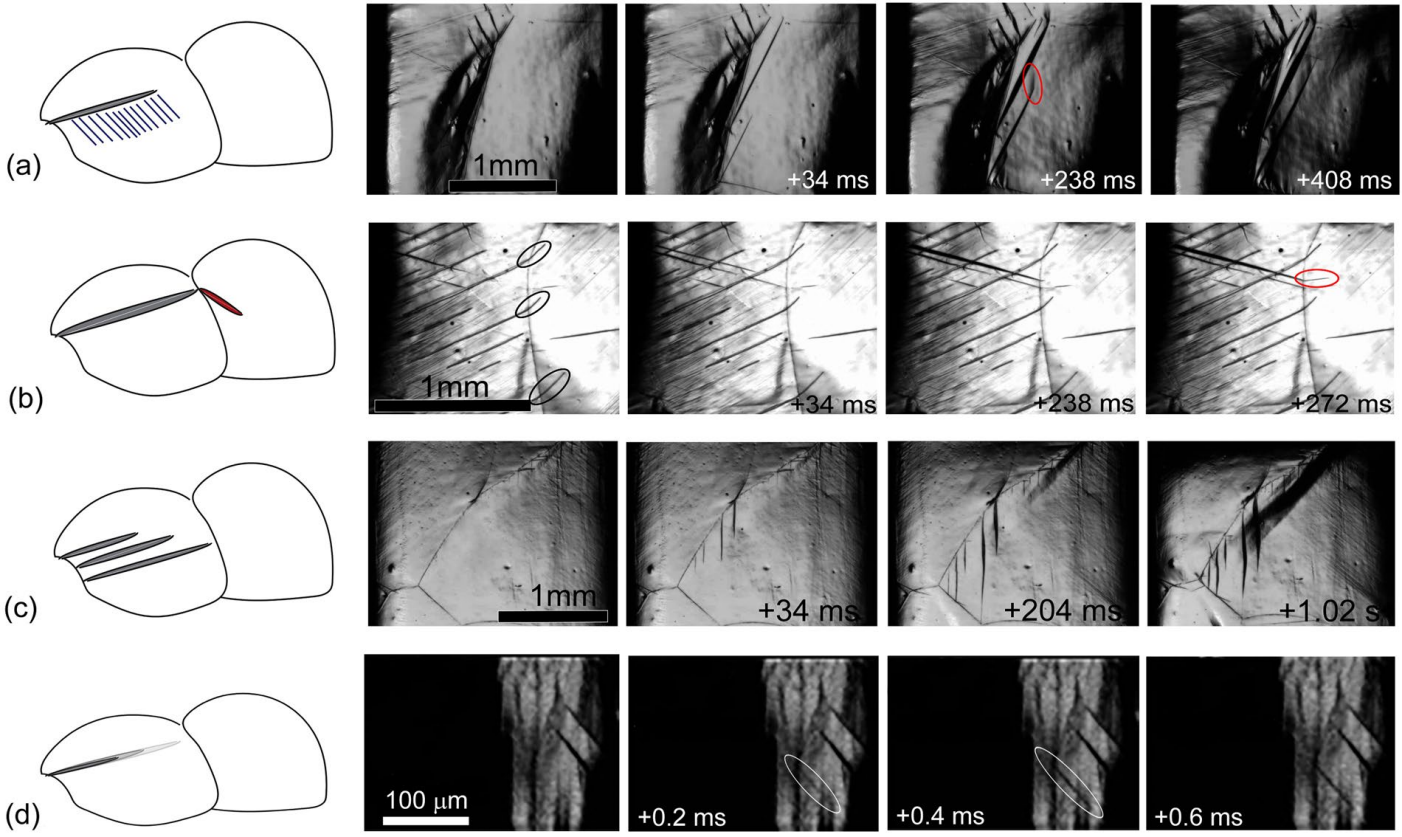
Schematic illustration of four possible mechanisms of temporal correlation in the twinning induced AE time series (**a**) the twin-dislocation interaction, (**b**) the relay-like mechanism whereby the lenticular twin terminating at the grain boundary promotes initiation of a new twin in the neighbouring grain, (**c**) concurrent propagation of several closely spaced twins in the same grain and (**d**) self-correlated propagation of the twin lamella. Consecutive snapshots obtained from *in situ* high-speed video recording of twinning related processes in Mg are shown on the right-hand side in support of the proposed mechanisms.

There is abundant literature regarding dislocation-twin and twin-twin interactions^58–61^, which helps to shed light on the observed temporal correlation between the twinning-induced events in AE time series. The possible reasons for that include (a) the twin-dislocation interaction revealing itself, for example, as the activation of dislocation slip ahead of the propagating twin^58,62^ to relieve the stresses that exist at the tip of the twin terminating within the crystal^63,64^ (Fig. 7a), (b) the relay-like “twin induced nucleation mechanism” (Fig. 7b) whereby the lenticular twin shoots through the grain, terminates at the grain boundary and promotes initiation of a new adjoined twin in the neighbouring grain due to the stress concentration at its tip^65–70^, (c) lengthwise propagation of twin bundles (Fig. 7c) whereby several closely spaced parallel twin variants extend nearly concurrently in the same parent grain^40^ (see also^71^ for the evidence of twin clustering) and (d) auto-correlated jerky motion of the twin front (Fig. 7d) controlled by the intermittent flow of twinning dislocations^72,73^. All of these scenarios are illustrated in Fig. 7 showing the consecutive snap-shots of the specimen surface obtained by *in situ* high-speed video recording of deformation processes in Mg (the experimental setup has been described in^74^). Although none of these three scenarios can be completely disregarded, the first three cannot be considered as leading contributors to the existing temporal correlations since their time scales do not match the relatively short characteristic times where the deviation of the observed process from the ideal Poisson distribution is observed in the AE signal. On the other hand, Fig. 7d illustrates that the thin twin lamella can advance rapidly in the longitudinal direction in the jerky “stop-and-go” manner with short waiting times between jumps. It has been demonstrated experimentally and argued theoretically^20^ that even a micrometre scale advance of the twin with the velocity being of the order of the speed of sound, or a sizable fraction of it, induces measurable transient AE signals.

As a matter of caution, one should bear in mind that all the above considerations regarding the random data analysis have been originally developed for stationary (homogeneous) processes. One could reasonably suspect that the observed deviations of inter-arrival times from the Poisson distribution may not necessarily be caused by the longer memory of the past but might occur as a result of non-stationarity of the process at different points. Strictly speaking, the AE time series presented in Fig. 1, as well as other similar data we have obtained so far on Mg alloys, cannot be regarded as a “perfectly” stationary stochastic process, i.e. as a process whose unconditional joint probability distribution does not change in time. However, it can be considered as a weakly or “quasi-stationary” process on the time scale of individual realisations used for the analysis. Nevertheless, to make sure that the non-stationarity associated with varying AE activity cannot be the reason for the observed features, the same algorithm of signal detection and processing was applied to the synthetic time series simulating the strongly inhomogeneous Poisson process with the intensity $\lambda (t)$ changing from 0 to 600 s^−1^ within 20 s according to the sine function. In all cases tested, the proposed algorithm correctly identifies the process as the non-stationary Poisson one even though the variation in λ was much stronger than that measured experimentally in our tests. Thus, the observed AE behaviour cannot be attributed to the non-stationarity of the slowly evolving time-series.

## Summary and Conclusions

The results of the present work provide new insights into the physics of the twinning generation process and the temporal evolution of plasticity in twinning-prone materials through the proposed quantitative statistical analysis of the AE time series. The most intriguing result is that unlike dislocation slip, the mechanical twinning falls into a class of non-Poisson processes, i.e. it manifests itself as a process with a memory of the past. The AE time series were analysed statistically with independent methods from different angles, aiming at revealing fine features in the temporal dynamics of the underlying processes. The innovative threshold-less detection method was applied to determine the arrival times of events constituting the AE point process. The temporal event flow structure was described by the distribution of time intervals between successive events. Combining this analysis with the advanced adaptive sequential *k*-means signal categorisation technique, the predominant contributions of both dislocation slip and twinning were separated in the AE signal. The dislocation slip appeared as the random Poisson process with the exponential distribution of waiting times between the points. As opposes to this, it was undoubtedly demonstrated that the mechanical twinning in Mg alloys did not belong to the same class. Rather it can be approximated by the Hawkes self-excited process with the short memory of the past being of the order of 1 ms or less, and corresponding to the auto-correlated lengthwise propagation of the twin front.

As the additional outcome of this work, it can be concluded that the AE, when powered by modern signal detection, characterization and classification techniques, opens up a way to characterize the intimate details of the evolution of the dislocation and twin sub-systems quantitatively. While each of the two proposed analytical methods - statistical analysis of the inter-arrival times and adaptive categorization of spectral density functions - taken in isolation offers access to the dynamics of the AE sources on different scales, employing them together yields a much more conclusive picture.

As a final note, we should notice that the observed non-Poisson type behaviour of the twinning–related AE sources during tensile deformation of the coarse grain alloy ZK60 is not specific to the alloy, its grain structure or the deformation mode. Although in the present work the conclusions were supported by the results obtained in monotonic tension of the specific alloy, essentially the same AE behaviour was ubiquitously observed whenever the twinning was a dominant deformation mode in pure Mg, Ti, Zn or in their alloys with different grain sizes subjected to either tensile, compressive of cyclic deformation.

## Materials and Methods

### Materials and testing

Commercial magnesium alloy ZK60 (Mg-5.8Zn-0.44Zr, in wt.%) with the average grain size of 70 ± 13 μm after hot pressing was used as a representative of materials prone to twinning. Further details of its microstructural characteristics can be found in^7,75^. For the sake of comparison between the materials exhibiting twining and materials with dislocation-mediated plasticity, a plate of high purity 99.99% α-Iron produced by Nilaco Corporation (Japan) was two-step annealed at 1250 °C and 900 °C for 1 h per step in vacuum to obtain a uniform microstructure with random texture and grain size of 100 ± 30 μm. Mechanical tests were conducted on flat dog-bone shaped specimens having the gauge dimensions of 10 × 4 × 3 mm^3^. The specimens from both metals were shaped by an electric wire cutter and mechanically polished to a mirror-like finish. They were tensile tested to failure with a nominal strain rate of 5 × 10^−3^ s^−1^. A miniature broadband AE sensor PICO was mounted securely on an unstrained shoulder part in close proximity to the gauge part of the specimen. Vacuum oil was used as a coupling medium to ensure efficient transfer of elastic waves from the surface to the transducer. The signal was amplified by 60 dB in the frequency band from 50 to 1200 kHz and transferred to the AE data acquisition system based on a 16 bits PCI-2 (Physical Acoustics Corp., USA) board. AE recording was performed continuously in a threshold-less mode at a sampling rate of 2 MHz.

### Data processing

Data processing includes the following steps:

(i) signal detection and arrival time picking;
(ii) analysis of the arrival time statistics by calculating the probability density function and performing its Chi-square goodness-of-fit test for the Poisson distribution;
(iii) independent Bi-test for the same purpose;
(iv) non-supervised signal classification by the Adaptive Sequential *K*-means (ASK) clustering algorithm aiming at the discrimination between the signals mediated by dislocation slip and twinning;
(v) modelling the non-Poissonian time-series by a Hawkes type self-exciting process and its statistical verification vis à vis experimental data, using the random time change theorem.

#### Signal detection

To convert the continuously recorded AE data, Fig. 1a, into a sequence of arrival times, the signal detecting algorithm proposed in^76^ (see also^77^ for details) was used. The sliding-window technique employed a time window of 4096 samples moving by 4 samples through the whole raw dataset. For each signal fragment, a Fourier power spectral density (PSD) function $G(f)$ was calculated using a periodogram technique. The signal detector ϕ is constructed as:

5 $$\phi (t)=\frac{1}{{\rm{\Delta }}f}{\int }_{{f}_{{\rm{\min }}}}^{{f}_{{\rm{\max }}}}[\frac{\partial \,\mathrm{ln}\,G(f,t)}{\partial t}]df$$

with ${\rm{\Delta }}f={f}_{{\rm{\max }}}-{f}_{{\rm{\min }}}$ - the frequency band. As long as the process is stationary, e.g., when no AE appears, and the recorded signal is due to noise, its spectral density does not change, $G(f,t)=const$ and $\phi (t)=0$. However, as soon as the burst AE signal arises and $G(f,t)$ changes correspondingly, $\phi (t)\ne 0$ and the signal is detected, Fig. 1c (inset). Due to the derivative nature of $\phi (t)$, it is extremely sensitive even to the very subtle transient signals buried into the stationary background noise. To implement this algorithm, the raw streaming data were divided into *K* successive realisations of duration *T*, Fig. 1d, which is chosen to be long enough to ensure a sufficiently large number of events detected. For each *j-th* realisation, the ϕ-transform detects ${N}_{j}$ readings of arrival times ${t}_{j}$. Finally, a complete set of arrival times $\{{t}_{0},{t}_{1}\,\mathrm{...}{t}_{i}\,\mathrm{...}\,{t}_{N}\}$ was obtained for the whole dataset.

#### Analysis of arrival times statistics

Since the properties of any random process are fully determined by the probability density function (PDF) of a descriptive random variable, the PDF $\rho ({\rm{\Delta }}t)$ is obtained for each *j-th* realisation, and the statistical goodness-of-fit χ^2^ (Chi-square) test is applied to probe the agreement between the inter-arrival times Δ*t* and the Poisson distribution according to Eq. (2). Note that for a Chi-square goodness of fit test, the null hypothesis states that the data are consistent with a specified distribution (Poisson distribution of inter-arrival times in our case). The alternative hypothesis is that the data are not consistent with the Poisson distribution. The null hypothesis is rejected if the computed Chi-square value is greater than the table critical value at a given level of significance. The process intensity (count rate) entering Eq. (1) is computed as ${\lambda }_{j}={N}_{j}/T$ for each realisation $j\in [1,K]$.

#### Bi-test

The advantage of this test is that it is applicable to strongly non-stationary processes. For each *t*_*k*_ event, a value ${B}_{k}=\frac{{{\rm{\Delta }}}_{k}}{{{\rm{\Delta }}}_{k}+{\delta }_{k}/2}$ is calculated^47,48^, where ${{\rm{\Delta }}}_{k}=\,{\rm{\min }}\,[{t}_{k}-{t}_{k-1};{t}_{k+1}-{t}_{k}]$ is the time distance to the nearest event, and $${\delta }_{k}=\{\begin{array}{c}{t}_{k-1}-{t}_{k-2}\,if{{\rm{\Delta }}}_{k}={t}_{k}-{t}_{k-1}\\ {t}_{k+2}-{t}_{k+1}\,if\,{{\rm{\Delta }}}_{k}={t}_{k+1}-{t}_{k}\end{array}$$ is the time distance to the second successive event. If the AE process obeys a local Poissonian behaviour, the variable *B* should be evenly distributed between 0 and 1 with the mean $\langle B\rangle =1/2$. The deviation of the experimental cumulative distribution function *F(B*_*k*_) from the theoretical uniform distribution according to the Kolmogorov-Smirnov test with the 95% confidence interval indicates the event-event correlations.

#### Cluster Analysis

Mathematical details of this procedure are discussed in^6^ along with the benefits of this algorithm. The two most important features should be emphasised: (a) the number of clusters to be derived from a dataset is not specified *a priori* but is ‘data-driven’, and (b) the process is non-iterative, i.e. the AE signals are associated with a certain cluster sequentially as they arrive one after another. The procedure starts with an evaluation of the power spectrum of a background noise, which serves as a first reference point for comparison and the first cluster creation^6^. The symmetrised Kullback-Leibler divergence ${d}_{KL}$ was chosen as a measure of pair-wise similarity/dissimilarity between normalised discrete PSDs ${G^{\prime} }_{i}(f)\,$ and ${G^{\prime} }_{j}(f)\,$^6^:

6 $${d}_{KL}=\sum _{m\,=1}^{N}({G^{\prime} }_{im}-{G^{\prime} }_{jm})\mathrm{log}(\frac{{G^{\prime} }_{im}}{{G^{\prime} }_{jm}})\,$$

Each signal is either assigned to the nearest cluster or is used as a seed of a new cluster. The algorithm tends to minimise intra-cluster distances and maximise inter-cluster distances.

#### Model verification (the goodness of the model fit testing)

When a candidate model for the process studied is considered (e.g. Pólya, Hawkes, Cox, or, etc.), it is necessary to check it against experimental data to verify how good it is. In statistics, the *random time change theorem*^78^ is often used for this purpose. This theorem is an important result of martingale-based point process theory demonstrating a fundamental possibility to transform a wide class of point processes into a homogeneous Poisson process. It states that if *t*_*1*_, *t*_*2*_, *… t*_*n*_ (${t}_{i}\in [0,T],\forall i$) is a realisation from a point process with the positive conditional intensity function ${\lambda }^{\ast }{(t)}_{{H}_{t}}$ and if the function

7 $${\rm{\Lambda }}(t)={\int }_{0}^{t}{\lambda }^{\ast }{(s)}_{{H}_{t}}ds$$

is strictly ascending and ${\rm{\Lambda }}(t) < \infty$, then the transformed time-series $\{{t}_{1}^{\ast },{t}_{2}^{\ast }\,\mathrm{...},{t}_{n}^{\ast }\}=\{{\rm{\Lambda }}({t}_{1}),{\rm{\Lambda }}({t}_{2})\,\mathrm{...},{\rm{\Lambda }}({t}_{n})\}$ called a *residual (*or *rescaled residual) process* form the Poisson process with unit rate^55,79^. In other words, the distribution of rescaled time-intervals $\{{\tau }_{1}^{\ast },{\tau }_{2}^{\ast },{\tau }_{3}^{\ast }\}=\{{t}_{1}^{\ast },{t}_{2}^{\ast }-{t}_{1}^{\ast },{t}_{3}^{\ast }-{t}_{2}^{\ast }\mathrm{..}\}$ should obey the exponential distribution with unit mean intensity $\lambda =1$ and probability density $f({\tau }^{\ast })=\exp (\,-\,{\tau }^{\ast })$. This theorem yields a powerful means for both the diagnostics for point process models and/or simulation of various point processes. Since the point process is defined by the conditional intensity function:

8 $${\lambda }^{\ast }{(t)}_{{H}_{t}}=f{(t,{\rm{\Theta }})}_{{H}_{t}}$$

which is a function of time *t* and the set of parameters Θ specific for a given model, c.f. Eq. (4) for the Hawkes model, it is plausible to start from the numerical computation of model parameters Θ_opt_ approximating the experimental count rate $\lambda (t)$ using the Nelder-Mead simplex optimization method^80^. Optimal parameters Θ_opt_ are then used to compute the expected model intensity function ${\lambda }^{\ast }{(t)}_{{H}_{t}}$. Finally, the goodness of the model fit is verified on the basis of the random time change theorem with an aid from the following four independent statistical procedures which are commonly used to test the suitability of the candidate model.

(i) The quantile of the ${\tau }^{\ast }$ distribution is plotted against the quantile of the $\exp (-{\tau }^{\ast })$ distribution for visual evaluation whether the two data sets come from populations with a common distribution.
(ii) Transforming the intervals ${\tau }_{i}^{\ast }$ once again by applying the following transform
  9 $${U}_{i}=1-\,\exp (-{\tau }_{i}^{\ast }),$$
  one should obtain a set of uniformly distributed variables $U\in [0,1]$. The mutual independence of variables *U* can be visually judged from the *U*_*i*_ vs *U*_*i−1*_ scatter plot. If the intervals ${\tau }_{i}^{\ast }$ are independent over the whole definition range, the uniform distribution of points is supposed to be seen. The irregular patterns such as dense clusters of points would assume a memory of the past or some sort of correlation between the events in the initial process.
(iii) The CDF for the uniformly distributed variables *U*_*i*_ should be a straight line.
(iv) The ${\chi }^{2}$ Pearson’s criterion can be used for quantitative comparison of the goodness of agreement between experimental inter-arrival times ${\tau }_{i}^{\ast }$ and the theoretical distribution $\exp (\,-\,{\tau }^{\ast })$.
